# Extraosseous Benign Notochordal Cell Tumor Originating in the Lung: A Case Report

**DOI:** 10.1097/MD.0000000000000366

**Published:** 2015-01-09

**Authors:** Yusuke Takahashi, Toru Motoi, Masahiko Harada, Yumiko Fukuda, Tsunekazu Hishima, Hirotoshi Horio

**Affiliations:** From the Department of Thoracic Surgery (YT, MH, HH); and Department of Pathology (TM, YF, TH), Tokyo Metropolitan Cancer and Infectious Diseases Center, Komagome Hospital, Bunkyo-ku, Tokyo, Japan.

## Abstract

Benign notochordal cell tumors (BNCTs) are tumors originating in the axial skeleton, where chordomas occur. Although very rare, some cases of extraosseous chordoma, such as in the soft tissue and lungs, have been reported. We report a case of a primary tumor showing the notochordal characteristics of BNCTs within the axial skeleton.

An asymptomatic 57-year-old woman presented with an abnormal shadow on her chest radiograph; chest computed tomography revealed a well-defined round nodule. The resected sample tissue contained a jelly-like small nodule. Histologically, it was identified as a BNCT, based on minimal nuclear atypia, extremely low mitotic activity within the tumor cells lying in a sheet-like arrangement, and focal immunopositivity for brachyury.

This is the third case report of BNCT originating in the lungs; BNCTs are considered asymptomatic tumors that are identified by using highly developed chest imaging technology; however, our findings also suggest that these notochordal tumors may potentially originate from extraosseous sites that lack ideal precursor cells. Our case suggests that notochordal tumors can arise from organs that are unrelated to known notochordal development.

## INTRODUCTION

Benign notochordal cell tumors (BNCTs) are tumors that originate from notochordal cell remnants,^1^ and they have been recently identified as a benign counterpart and possible precursor lesion of chordomas.^2^ Notochordal tumors typically originate in the axial skeleton, where notochordal remnants exist; however, a few extremely rare cases of notochordal tumors have been shown to arise primarily from the soft tissue and visceral organs.^3^ Interestingly, to date, organ-derived notochordal tumors are only confined to the lungs; the reason for this finding remains to be elucidated because of the rarity of the tumor. Herein, we report a case of extraosseous BNCT originating in the lung that was confirmed by comprehensive pathological findings and the immunohistochemical expression of brachyury, a reliable marker for notochordal tumors.

## CASE REPORT

**FIGURE 1:**
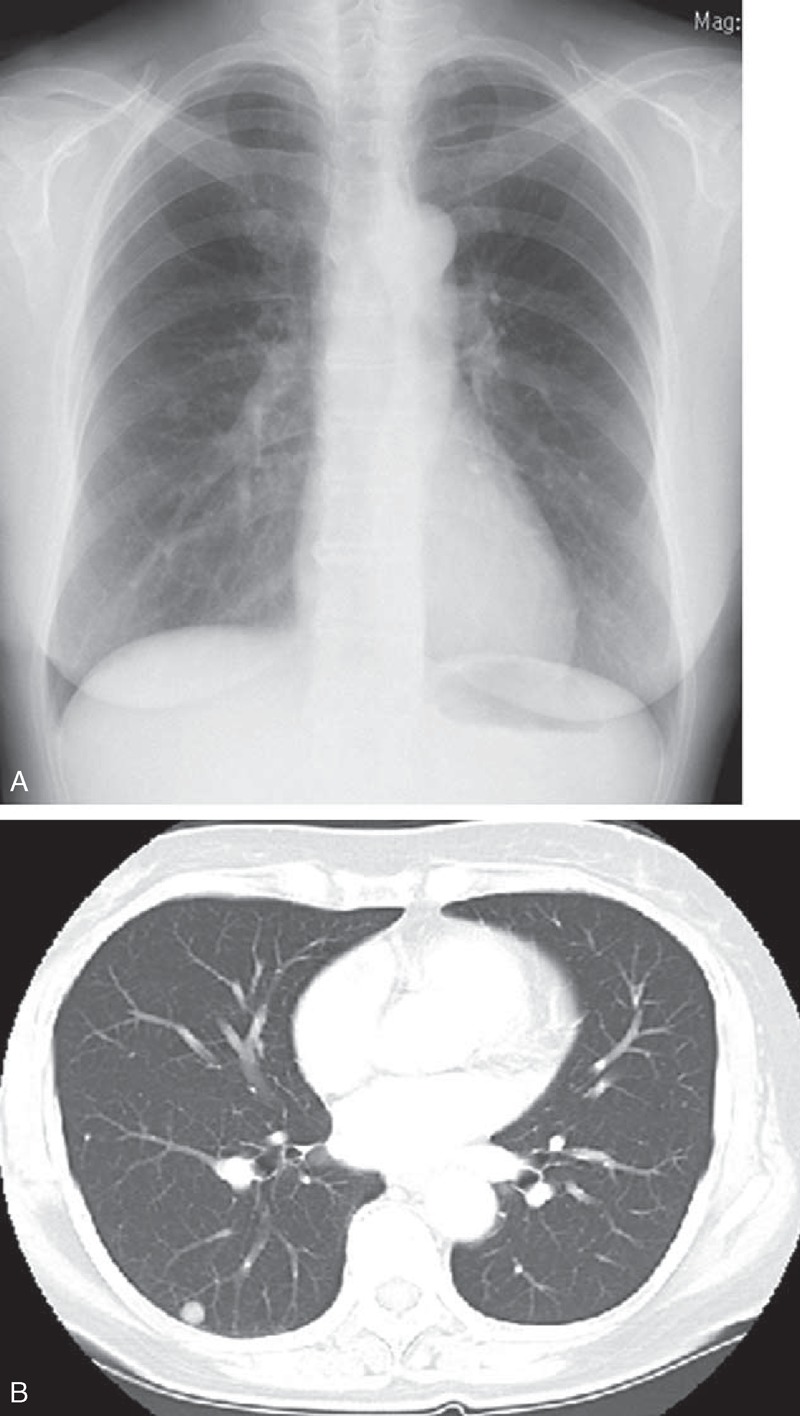
(A) Chest radiograph upon admission to our hospital showing a well-defined nodular shadow in the right middle lung field. (B) Chest computed tomography scan showing a well-defined, round, solid nodule of 10 mm size, located in the right segment 6.

A 57-year-old, asymptomatic woman with no history of smoking was referred to our hospital after a chest radiograph revealed a nodular shadow in the right middle lung field (Figure 1A). Chest computed tomography (CT) showed a well-defined, round, solid nodule, which was 10 mm in diameter, in segment 6 of the right lung (Figure 1B). Positron emission tomography/CT did not demonstrate any other lesions in other parts, including the bones or the soft tissue. Physical and laboratory examinations revealed no significant abnormalities. Furthermore, the levels of serum tumor markers were not elevated. Imaging analysis revealed that the pulmonary nodule was adjacent to the visceral pleura; however, it was difficult to use the endobronchial approach to obtain adequate samples for histological diagnosis. Therefore, we performed thoracoscopic wedge resection of the right lower lobe for diagnostic purposes; no other pulmonary nodules were found intraoperatively.

**FIGURE 2:**
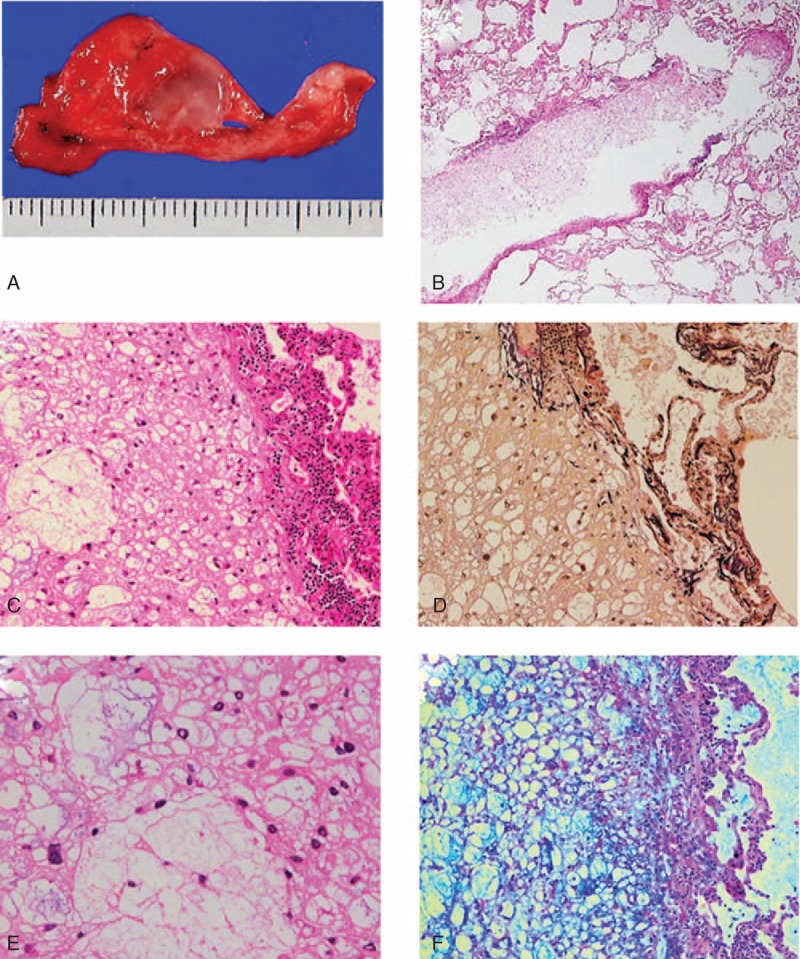
(A) Macroscopically, a jelly-like tumor is located in the lung parenchyme. (B) A paucicellular and avascular tumor with cuffs of inflammatory cells observed in the alveolar space. (C) Multivacuolated tumor cells with microcytic change and lymphoid cuffs are visible. (D) The tumor shows minimal destruction of the alveolar structure (elastic Van-Gieson staining). (E) The nuclei of the tumor cells show minimal atypia, with focal and mild pleomorphism (hematoxylin and eosin staining). (F) Intracellular and intracystic mucin accumulation is noted (alcian-blue/periodic acid–Schiff staining).

Macroscopically, the resected sample contained a homogeneous, jelly-like transparent tumor, 10 mm in maximum diameter, in the lung parenchyma (Figure 2A). Histologically, the tumor was well demarcated and paucicellular and it consisted of large tumor cells with a low nuclear/cytoplasmic ratio and prominent intracellular vacuolation (Figure 2B). The tumor, which lacked a fibrous stroma, was also devoid of blood and lymphatic vessels. Instead, the tumor cells had proliferated in a prominent myxoid background. Cuffs of lymphocytes and minimal destruction of the alveolar structure were observed at the periphery of the tumor (Figure 2C and D). Adipocyte-like, univacuolated, and multivacuolated cells were intermingled within the tumor; the latter resembled physaliphorous cells that are typically observed in chordomas. Moreover, most of the tumor cell nuclei were small and round, with fine chromatin and less conspicuous nucleoli. However, some of the tumor cells were irregular in shape and had larger nuclei (Figure 2E); neither mitotic figures nor vascular invasion was observed. A myxoid substance was observed in the cytoplasm of vacuolated tumor cells and microcystic space on alcian-blue staining, which also stained the adjacent alveolar spaces (Figure 2F).

**FIGURE 3:**
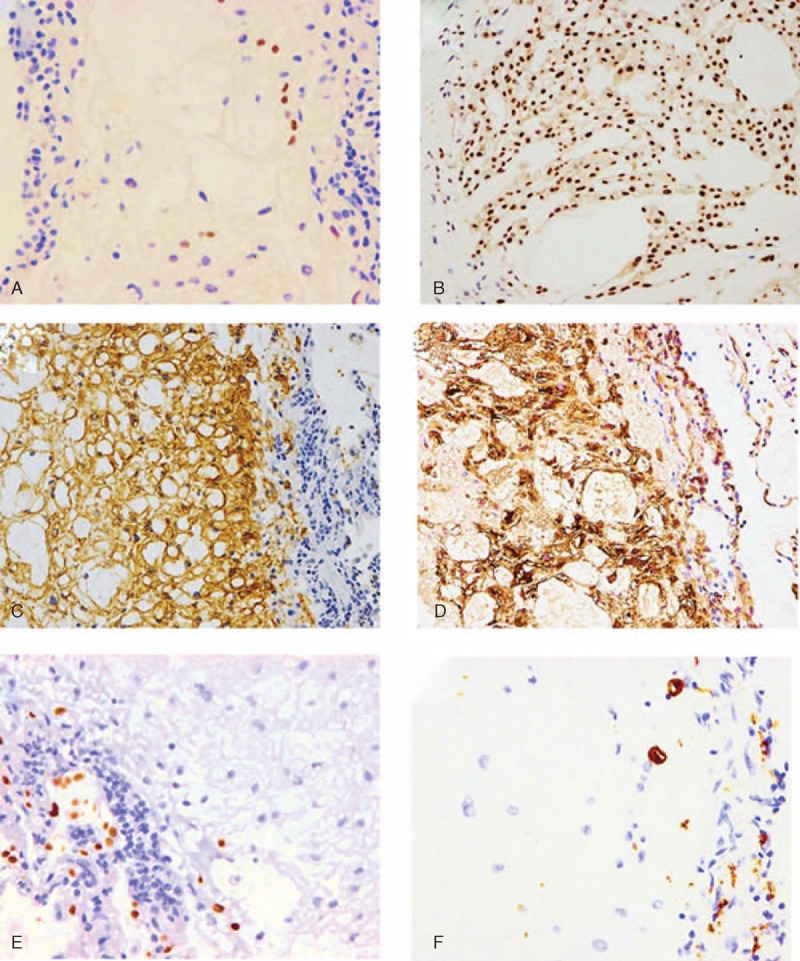
(A) Brachyury immunostaining showing focal expression and brachyury-positive expression in the tumor cell nuclei. (B) Referenced conventional axial chordomas showing diffuse brachyury-positive expression in the nucleus. (C) Similar to axial benign notochordal cell tumors, tumor cells showing diffuse positive expression for epithelial markers including cytokeratin AE1/AE3. (D) Vimentin expression is diffusely positive in tumor cells. (E) Positive expression of thyroid transcription factor-1 is only visible in pneumocytes entrapped at the margin of the tumor. (F) Cluster of differentiation 68 expression was completely negative in tumor cells, but infiltration in the alveolar macrophages at the periphery can be observed.

On immunohistochemical staining, brachyury, a highly specific diagnostic marker of notochordal differentiation, was focally but undoubtedly positive in the nuclei of the tumor cells (Figure 3A). We compared the ratio of the brachyury-positive cells from the present case with 3 other conventional chordomas of the axial bone by counting 100 nuclei. This analysis was approved by the Institutional Ethical Committee (KH-1460) and the written informed consent was obtained from the patients. The results showed that the ratio of brachyury-positive cells in the present case (14%) was much lower than that of the control chordoma cases (90%–98%, mean 93.3%) (Figure 3B). The tumor was also positive for immunohistochemical markers for chordomas and BNCTs arising from the axial skeleton, including cytokeratins (CKs) AE1/AE3 (pan CK), CAM5.2 (low-molecular-weight CK), vimentin, S100 protein, CK7, CK18, and CK19 (Figure 3C and D). Conversely, the tumor cells were negative for CK5/6, CK20, thyroid transcription factor-1, napsin A, cluster of differentiation (CD) 68, and CD163 (Figure 3E and F). The ratio of MIB1 (Ki-67)-positive cells within the tumor was extremely low (2.2%).

In order to exclude the possibility of the lung tumor having metastasized from an unknown primary site, an extensive radiographic examination that included whole body CT, magnetic resonance imaging of the whole spine, and bone scintigraphy was performed. The results showed an absence of tumors throughout the body. Five years after surgery, the patient is alive without tumor recurrence or metastasis.

## DISCUSSION

Notochordal tumors, which comprise both chordomas and BNCTs, are generally considered to originate from notochordal remnants.^1^ Chordomas are malignant tumors that account for approximately 1% of primary malignant bone tumors, with an incidence of 0.08 per 100,000 people.^4^ In contrast, BNCTs are benign tumors of notochordal origin whose distribution is similar to that of chordomas; however, their incidence is still unknown.^5^ BNCTs tend to present as small tumors, measuring approximately 4 mm in size.^5^ Moreover, cases of coexistent chordomas and BNCTs have been reported, suggesting the sequential progression of BNCTs to chordomas.^6^

Although rare, chordomas may arise from extra-axial locations, which are mostly of soft tissue origin.^3,7,8^ Besides soft tissue, there are a few reports of chordomas that originate in the lungs, which is currently the only reported visceral organ where extraosseous chordomas occur.^9,10^ Interestingly, Kikuchi et al^11^ recently reported 2 cases of incidental notochordal tumors of the lung that were indicative of BNCTs, as evidenced by brachyury expression. Furthermore, Lee et al^12^ reported an additional case of BNCTs in both the lungs. The histological and immunohistological findings from our case are quite similar to the previously reported tumors, except for the lack of a central cyst formation in our case. Of note, the tumor in this case showed mucin production as evidenced by alcian-blue staining, which is an uncommon finding in typical BNCTs, but not in chordomas. However, destruction of the alveolar structure, nuclear atypia, and mitotic activity of the tumor cells remained minimal, suggesting the benign nature of the tumor in this case. The histological and immunohistochemical profiles of the 3 lung BNCTs, including ours, revealed almost identical characteristics with BNCTs from the axial skeleton and showed brachyury expression. In contrast, no soft-tissue BNCTs have been reported thus far. This may be partly explained by the use of highly developed chest imaging technologies that can detect incidental small tumors such as BNCTs, rather than the fact that the lung is considered a specific source of notochordal tumors.

Brachyury has been identified as a key transcription factor in the early phase of posterior mesoderm development, which includes the development of hemangioblasts and the notochord.^3,13,14^ Brachyury was recently established as a highly sensitive and specific immunohistochemical marker of notochordal differentiation, and it has enabled the accurate diagnosis of notochordal tumors by differentiating them clearly from other histological mimics.^11–13^ Although the tumor was brachyury positive in our case, the positive signal was only focal. As almost all chordoma tumor cells are reported to be diffusely positive for brachyury, we compared the ratio of brachyury-positive cells of our tumor with that of 3 conventional chordomas. The ratio of cells that were brachyury positive was dramatically lower in our case than the referenced chordomas. To the best of our knowledge, no other study has analyzed the differences in brachyury-positive expression between BNCTs and chordomas. However, it should be noted that brachyury-positive immunostaining was limited to focal expression, at least in a subset of BNCTs. It is unclear whether this phenomenon is specific for this case or is a common characteristic of all BNCTs. Further studies will need to be conducted to confirm this expression pattern of brachyury in notochordal tumors.

After excluding the possibility of a metastatic tumor, differential diagnosis of the primary tumor included several possible types of tumors: chordoma, extraskeletal myxoid chondrosarcoma, myoepithelioma/mixed tumor, benign peripheral nerve sheath tumor showing myxoid change, chondrosarcoma, myxoid liposarcoma, primary pulmonary myxoid sarcoma with EWSR1–CREB1 fusion, pleomorphic adenoma, chordoid meningioma, and mucinous carcinoma. Based on the histopathological diagnostic analysis, malignant tumors were excluded by the characteristic benign appearance, which included minimal destruction of the alveolar structure, absence of nuclear atypia, and extremely low mitotic activity of the tumor cells. Moreover, the tumor was devoid of expression of other specific immunohistochemical markers, with the exception of brachyury, which is highly specific marker for chordoma and BNCT. Indeed, the tumor cells in our study were almost identical in histological appearance to BNCT of bones.

In agreement with the previous reports, BNCTs of the lung are benign tumors that are detected on imaging examinations; however, it is still unknown whether they can be considered as precursors of chordomas. Moreover, it may be difficult to differentiate these tumors from other primary and metastatic myxoid tumors, based solely on imaging analyses. Therefore, tumor resection followed by brachyury detection for pathological diagnosis may be appropriate for treatment. Furthermore, the existence of primary BNCTs of the lung suggests that notochordal tumors can arise from organs that are unrelated to known notochordal development.
